# The Politicised Child, Transcultural Constructions of Childhood, Psychological Trauma, and the Mind in the Modern World: Afterword

**DOI:** 10.1007/s11013-022-09804-2

**Published:** 2022-08-13

**Authors:** Derek Summerfield

**Affiliations:** Institute of Psychiatry, Psychology and Neuroscience, London, UK

Ana Antic notes in her Introduction that this edition addresses the public emergence of the harmed (‘traumatised’) or politically tainted child—shades of Kipling’s ‘half devil, half child’ or, here, Wagner’s ‘white child gone bankrupt’. Souls to be saved which would otherwise be ‘lost’. These essays seek to interrogate the subject across disciplines and domains: social history, social anthropology, psychology, humanitarian and human rights discourses. In part this is a critique of the conceptual assumptions and limitations of Western scholarship, particularly so in relation to psychology, whose universalist and biomedical underpinnings present a barrier to nuance and complexity.

I would like to reflect on 3 key poles. Firstly, what is ‘psychology’? Is it only what the clinical psychology profession says it is? Is it a moral philosophy of the self? Is the business of other people’s minds more a matter of philosophy than of science, which cannot separate fact from value? Can ‘psychology’ be distinct from ‘culture’? From an anthropological point of view, what psychology invokes includes personal identity, selfhood, subjectivity, memory, consciousness, emotion, motivation, cognition, madness, and ‘mental health’. In modern Western society the backdrop is of the medicalisation of everyday life, the commodification of ‘mind’, neoliberal injunctions towards self-improvement, and the insertion into public consciousness of the language of psychological deficit.

How far does it take us to use the Western ethnopsychological definition of ‘emotion’ as: internal, often biological, unintended, distinct from cognition, and a feature of individuals not situations? There are non-Western tribal societies who do not recognise our separation between ‘emotion and ‘cognition’- though in fact the feminist pioneer Mary Wollstonecroft made this point 200 years ago. However we witness the rise to iconic cultural status of ‘emotion’ and of ‘emotional trauma’, and a burgeoning industry to serve it. I do wonder if we are reaching a limit to the emancipation which from Freud onwards has seemed the promise of emotional expression and re-working. With this emphasis on introspection, self-pity and on pathologised, individual victimhood, have we made a turn into a cultural cul-de-sac? I would commend as one of the most swingeing and persuasive critiques of this: *We’ve had a Hundred Years of Psychotherapy- and the World’s Getting Worse* (1992) by James Hillman, a US Jungian psychologist.

This connects to what Stacey Hynd (2021) calls ‘affective truths’, which carry assumptions about authenticity, entitlement, redemption, and calls for compensation and justice, and which demand acceptance. These trade on the mobilisation of memory as a moral act. Indeed ‘memory’ has come to be the repository of the modern soul. But social documenters and historians cannot but be aware that the contents of memory are seldom unearthed in pristine condition, being shaped by subsequent events and the purpose to which the act of memorising is to be put. The narrative proffered by an unaccompanied Afghan minor in UK, or elsewhere by, say, a former child soldier, or a ‘white child gone bankrupt’, is not definitive, and might sound rather different if the conversation was, say, with another teenager or in some other setting. Such conversations might in part be less ‘Western’, might include references to vengeful spirits or displeased ancestors, reflecting cultural practices carrying externalised attributions for adversity. I would add that we tend to see exposure to particular violent or horrifying events as the ‘trauma’, but not a life of hunger, poverty and lack of any viable opportunities. Kirrily Pells notes that idioms of distress in Quechua people in Peru may relate not only to recent direct violence but to the symbolic violence of poverty, racism and lack of rights embedded in the colonial period.

When we opt for a conceptual distinction between what is ‘mental health’ and what is ‘social’ we are reproducing the tradition since the Enlightenment to regard the physical confines of the human individual as the basic unit of study, and for the mind to be examined by a technical methodology akin to that applied to the body, The mind, or ‘psychology’, is to be located inside the body- between the ears- whereas what is ‘social’ is outside the body, and outside the frame of reference. But it is more realistic to see our psychology in a Heideggerian sense, as playing out in the field upon which we move, and to consider the meaning of things- in particular a sense of coherence- as arising from our practical engagement with the world. Western mental health models can be half-blind to the role of social agency.

Secondly, our assumptions about human resilience. To speak aphoristically, the average citizen in a society will be as tough or as weak as society expects him or her to be. Yet one of the seminal shifts in the West in the twentieth century has been the rise of the idea that the average individual is not resilient but ‘vulnerable’, and likely in need of professional intervention, a shift described as cultural iatrogenesis by Ivan Illich in his brilliant and still prophetic study *Medical Nemesis* (1975). When statements that at any one time up to 1 in 4 of UK citizens have a mental disorder are regularly recycled in the media and are never contradicted by the Royal Colleges of Psychiatry or General Practice; when experiences of being bullied, or having a difficult labour, or performing the duties of a policeman or paramedic for which they are trained and paid, are seen as capable of causing non-brief mental consequences; when counselling teams are called automatically to a school where there has been a stabbing, in all these situations a different version of personhood is being posited. These emergent sensibilities now have a place in a Western context but would scarcely serve well in the harsh realities of most non-Western societies, where the premium must be on mere survival. The moral economy, the mentality that applies in societies of chronic scarcity, where the state delivers little and rights-based claims have little purchase, was beautifully articulated by a Darfur, Sudan traditional birth assistant who told a PhD student of mine: “Life is too short to worry too much, it is better to be satisfied with what is available.” Mentality is culture.

Thirdly, and connected to the broader cultural shift about resilience alluded to above, what is a ‘child’ or ‘childhood’ or an ‘adolescent’? It is often said that Western notions of a child reflect a ‘romantic’ nineteenth century invention, ‘innocent’ and a mere recipient of experience, and now propagated as a quintessentially social worker perspective. The children of the poor in eighteenth and nineteenth century UK undertook backbreaking work in harsh conditions in coal mines, and minors of 16 or younger were regularly subjected to public execution as if adults. In fact the last use of capital punishment on a teenager in UK was as recently as 1960. But public attitudes about what a child can put up with and recover from have altered even since then. 116 schoolchildren and 28 teachers were killed in the 1966 Aberfan, Wales, coal-tip disaster but the public aftermath was strikingly free of reference to psychological trauma, did not draw in formal psychological services, and newspaper accounts emphasised themes around stoic courage and endurance for survivors and bereaved families. There was an expectation of natural recovery, even from this. The situation would be starkly different today- though not of course in the many wars abroad where children serve as soldiers and are often proud of their contribution. Ismini Pells examines some of the questions around child agency and decision-making in desperate times.

There is the politicised child but also the obverse, the children whose lives and fate seemingly carry no political valence. The child shot dead by an Israeli soldier or settler in a polity in which, as the Israeli journalist Gideon Levy put it sadly in the newspaper Haaretz, “it is no big deal to kill a Palestinian child”. The Afghan children shredded by a bomb from a US drone when the algorithm used to decide targets mistakes a wedding party for a Taliban gathering. Street children in Brazil who are commonly characterised as “ham” or vermin, thus already extruded from society, and whose intermittent killing by off-duty policemen is approved of by middle class Brazilians. The children dying of kwashiorkor whom I saw, too late, as a rural government medical officer in the war in the last days of colonial rule in Rhodesia (now Zimbabwe). Their mothers wailed and threw themselves on the floor of the ward, but as far as the political order was concerned they died so ‘quietly’. To put it another way, if children’s lives are precious, which children? Perhaps a fundamental verity applies, part of what might be called an anthropology of ‘distance’: we retain our full sensibilities only in the local, amongst family, tribe, amongst people we know. Beyond that we are only occasionally engaged, prompted by an unusual event that captures our imagination, but even then not for long. The suffering of a family pet may mean more than that of children we do not know in places we do not know. An estimated 5 million (5 million!) children under the age of 5 died as a result of armed conflict in Africa in 1995–2015. What place do they have in the public record, these teeming near-ghosts, what traces of their weightless passage through the world? Can the social historian capture this?

Ana Antic asks whether humanities researchers can create accounts of the politicised child whose depth and fidelity serve not only the historical and anthropological record but also illuminate credible forms of assistance. The contributions in this edition show some of the ways ahead.
